# Inflammatory mediators and cartilage biomarkers in synovial fluid after a single inflammatory insult: a longitudinal experimental study

**DOI:** 10.1186/ar2640

**Published:** 2009-03-09

**Authors:** Janny C de Grauw, Chris HA van de Lest, Paul René van Weeren

**Affiliations:** 1 Department of Equine Sciences, Faculty of Veterinary Medicine, Utrecht University, Yalelaan 114, 3584 CM, Utrecht, The Netherlands; 2 Department of Biochemistry and Cell Biology, Faculty of Veterinary Medicine, Utrecht University, Yalelaan 2, 3584 CM, Utrecht, The Netherlands

## Abstract

**Introduction:**

Inflammation is an important feature of many joint diseases, and levels of cartilage biomarkers measured in synovial fluid may be influenced by local inflammatory status. Little is known about the magnitude and time course of inflammation-induced changes in cartilage tissue turnover as measured *in vivo *by synovial fluid markers. We aimed to study temporal changes in concentrations of inflammatory mediators, matrix metalloproteinase activity and cartilage biomarkers over 1 week in joints with experimentally induced inflammation.

**Methods:**

Localized inflammation was induced in the intercarpal joint of six horses by sterile injection of 0.5 ng lipopolysaccharide, and synovial fluid was collected at post-injection hours (PIH) 0, 8, 24 and 168. Concentrations of inflammatory mediators (prostaglandin E_2_, substance P, and bradykinin), general matrix metalloproteinase activity and markers of collagen II turnover (CPII and C2C) as well as aggrecan turnover (CS846 and glycosaminoglycans) were measured with appropriate assays. One-way analysis of variance on repeated measures was used to analyze differences in synovial fluid marker levels over time.

**Results:**

Lipopolysaccharide-injection led to a sharp rise in prostaglandin E_2 _at PIH 8, while substance P, bradykinin and matrix metalloproteinase activity showed more sustained increases at PIH 8 and 24. Glycosaminoglycan release paralleled changes in the CS846 epitope, with an increase by PIH 8, a peak at PIH 24, and return to baseline by PIH 168. For type II collagen, a parallel time course between catabolic (C2C) and anabolic (CPII) markers was also observed, but the time course differed from that seen for proteoglycan markers: collagen II markers peaked later, at PIH 24, and were still elevated over baseline at PIH 168.

**Conclusions:**

A primary intra-articular inflammatory insult, characterized by local release of peptide and lipid mediators and matrix metalloproteinase activation, can alter synovial fluid levels of proteoglycan biomarkers as early as 8 hours post-induction, and can lead to sustained rises in collagen II biomarkers during at least 1 week after onset.

## Introduction

Inflammation is an important factor in the pathogenesis and clinical presentation of equine joint disease as well as human joint disease [1-3]. Overt joint inflammation as may be seen in rheumatoid arthritis or infectious arthritis is known to have devastating effects on the joint tissues, most importantly the articular cartilage, and in many joint disorders local release of catabolic mediators plays an important role in the disruption of cartilage matrix homeostasis [4].

Intra-articular inflammation can be studied in more detail by means of analysis of proinflammatory cytokines, inflammatory mediators and catabolic enzymes in synovial fluid (SF). Articular cartilage damage, whether inflammatory or traumatic in origin, can likewise be studied indirectly by means of SF biomarkers of cartilage matrix turnover [5]. The extracellular matrix of articular cartilage is primarily made up of type II collagen and aggrecan. Biomarkers reflecting aggrecan as well as collagen II turnover have proven capable of signaling changes in cartilage matrix homeostasis in various disease states [5-10].

Of the many factors that may influence SF levels of cartilage turnover markers other than accumulated damage to the articular cartilage, local inflammation may be among the most important. Besides the potential dilution or washing-out of these markers due to joint effusion and/or altered clearance rates [11,12], there may be direct effects of inflammatory mediators and enzymes on articular cartilage turnover and thus on cartilage biomarker levels [13,14]. Few studies have so far attempted to quantify the effects of intra-articular inflammation on SF biomarker levels without the confounding effect of previous disease or concurrently created mechanical cartilage damage at baseline.

The horse is both a target animal for arthritis research and a suitable large animal model for the study of joint and cartilage disorders in humans [15-17]. In addition to having proportionately large joints from which ample SF can be obtained without the need for lavage or anesthesia [16], the horse is unique in that the pathophysiology of equine arthritis has been well studied for decades [18]. In horses, intra-articular lipopolysaccharide (LPS) injection in nanogram quantities is an established model for induction of transient localized sterile inflammation, which has been used to study clinical symptoms and gait parameters, drug pharmacokinetics, and/or the effects of therapeutic intervention [19-22].

The current study used this LPS model to investigate the influence of a single inflammatory insult on a panel of SF mediators and markers over the time course of 1 week. The panel included several inflammatory mediators and enzymes implicated in altered joint homeostasis in arthritic disease (prostaglandin E_2_, substance P, bradykinin, matrix metalloproteinase (MMP) activity), as well as turnover markers of aggrecan (chondroitin sulfate epitope 846 (CS846) and glycosaminoglycans (GAG)) and collagen II (carboxypropeptide of type II collagen (CPII) and collagenase-cleavage neoepitope of type II collagen (C2C)). We found that lipid and peptide inflammatory mediators and MMP activity show an early rise within 8 hours of induction of inflammation, with concomitant transient increases in aggrecan turnover markers within the first 24 hours. Collagen II turnover markers showed a similar parallel time course between catabolic and anabolic markers, but their response was delayed (starting at 24 hours), and persisted 1 week after induction of inflammation.

## Materials and methods

### Experimental animals

All experimental procedures and protocols were pre-approved by the Utrecht University Committee on the Care and Use of Experimental Animals in compliance with Dutch legislation on laboratory animal use. Six skeletally mature warmblood mares between 5 and 8 years of age with no history of orthopedic disease, free of lameness and with clinically and radiographically normal carpal joints were selected for this study. Horses were allowed a 2-week acclimatization period with once-daily hand-walking and were box-rested in separate 3.6 × 3.6 m^2 ^stalls on woodchip bedding for the duration of the experiment.

### Induction of inflammation

At post-injection hour (PIH) 0, one randomly assigned carpus of each horse was clipped and prepared for dorsal arthrocentesis. Lipopolysaccharide from *Escherichia coli *O55:B5 (catalogue number L5418, lot 057K4106; Sigma-Aldrich, St Louis, MO, USA) was diluted to a final concentration of 0.625 ng/ml in sterile lactated Ringer's solution. Horses were sedated with detomidine (0.01 mg/kg intravenously, Domosedan^®^; Pfizer, Capelle a/d IJssel, the Netherlands) and methadone (0.1 mg/kg intravenously; Eurovet Animal Health, Bladel, the Netherlands). Arthrocentesis was performed with a 21 G × 40 mm needle and 0.8 ml LPS solution was delivered aseptically into the intercarpal joint after withdrawal of the PIH 0 SF sample.

### Assessment of clinical outcomes

Before arthrocentesis at PIH 0, every 2 hours between PIH 2 and PIH 8, and at PIH 8, 24, 48 and 168, each horse's attitude, temperature, pulse and respiratory rate were recorded, lameness was scored on a standardized 0 to 5 scale [23], intercarpal joint effusion was graded on a scale from 0 to 4 as previously described [24], and carpal circumference was measured at the level of the accessory carpal bone with a tape measure. All scores were assigned and recorded by the same observer (JCdG).

### Collection of blood and synovial fluid

Blood was collected from the left jugular vein for routine hematology before sedation for arthrocentesis at PIH 0, 8, 24 and 168. Part of each SF sample was placed in ethylenediamine tetraacetic acid tubes for macroscopic evaluation, routine SF total white blood cell count with differentiation and total protein measurement (refractometer), while the remainder was centrifuged in plain tubes at 13,000 rpm for 15 minutes, aliquotted and stored at -80°C until further analysis.

### Synovial fluid mediator and marker analysis

A total of eight assays were performed on each SF sample. The prostaglandin E_2 _concentration was measured by a commercial ELISA (RnDsystems, Minneapolis, MN, USA) following RP-18 extraction of SF samples [25]. All peptide marker assays were performed in the presence of 1 mM (final concentration) phenylmethylsulphonylfluoride (an inhibitor of serine proteases). Substance P and bradykinin were measured using commercial enzyme immunoassay kits (substance P kit from Cayman Chemical, Ann Arbor, MI, USA; and bradykinin kit from Bachem, Bubendorff, Switzerland).

General MMP activity was measured by means of a fluorimetric assay based on cleavage of the fluorogenic peptide substrate FS-6 (Calbiochem, San Diego, CA, USA). This substrate was previously shown to be considerably more sensitive than FS-1 for measuring activity of collagenases (MMP1, MMP8, MMP13) in biological fluids [26]. In short, to 20 μl SF were added 80 μl MMP buffer (0.1 M Tris, 0.1 M NaCl, 10 mM CaCl_2_, 0.05% (w/v) Triton X-100, 0.1% (w/v) PEG6000, pH 7.5) and 100 μl of 10 μM FS-6 solution, after which the fluorescent signal was monitored for 10 minutes. The slope of the resultant linear curve (relative fluorescence units/second) was calculated as a measure of general MMP activity.

GAG release was quantified by the 1,9-dimethylmethyleneblue assay, adapted for use in microtiter plates [25]. Concentrations of the CS846 epitope (a putative marker of aggrecan synthesis [27,28]), as well as of CPII (a marker of type II collagen synthesis [29]) and C2C (a neoepitope present on collagenase-cleavage fragments of type II collagen [30,31]) were measured using commercial ELISA kits (IBEX, Montreal, Quebec, Canada). All of these assays were previously validated for use in the horse [25,32].

### Statistical analysis

Data are presented as the mean ± standard error of the mean. The effect of time after induction of inflammation on concentrations of SF parameters was tested by use of one-way analysis of variance on repeated measures. When a significant time effect was observed, levels at individual time points were compared with Tukey's *post hoc *tests. Categorical clinical variables were compared over time using a Friedman test, followed by Dunn's *post hoc *tests. Computer software was used (GraphPad Prism version 4.00 for Windows; GraphPad Software, San Diego, CA, USA) and the level of significance was set at *P *< 0.05.

## Results

### Clinical assessment

**Figure 1:**
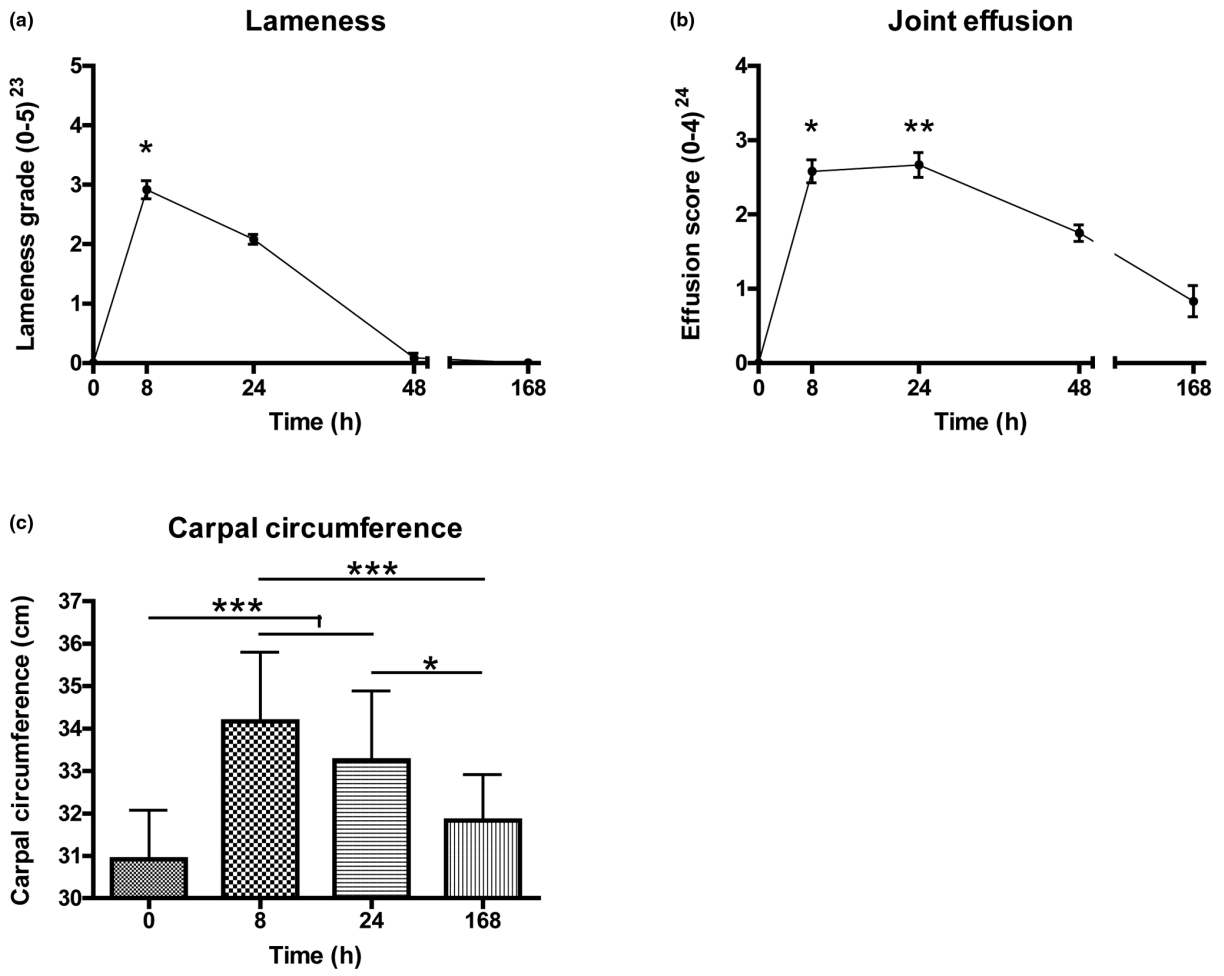
Clinical effects of lipopolysaccharide-induced joint inflammation. **(a) **Lameness grade, **(b) **effusion score and **(c) **carpal circumference following induction of synovitis in the intercarpal joints of horses (n = 6). Inflammation was induced at *t *= 0 by intra-articular injection of 0.5 ng *Escherichia coli *lipopolysaccharide. Data presented as the mean ± standard error of the mean. *P < 0.05, **P < 0.01, ***P < 0.001 compared with baseline (*t *= 0).

LPS injection led to a significant rise in lameness and effusion scores and carpal circumference (Figure 1). The lameness score was no longer significantly different from 0 at PIH 24 and lameness had resolved by PIH 48 in all six horses, while joint effusion showed a more gradual decline. No changes in appetite, pulse or respiration were observed, and rectal temperature and hematological variables remained within normal limits (data not shown).

### Conventional synovial fluid parameters

The results of routine SF analyses are presented in Table 1.

**Table 1:** Results of conventional synovial fluid analysis following intra-articular injection of 0.5 ng lipopolysaccharide

	Time after lipopolysaccharide			
	
	0 hours	8 hours	24 hours	168 hours
Total protein (g/dl)	1.27 ± 0.18	4.70* ± 0.18	4.80* ± 0.14	1.53 ± 0.18
Leucocytes (x10^9 ^cells/l)	0.25 ± 0.085	215.4* ± 15.9	64.6*,^† ^± 6.7	0.72 ± 0.38
Neutrophils (%)	N/A	98.8* ± 0.31	80.0*,^† ^± 3.4	N/A

Comparison of synovial fluid total protein, white blood cell counts and differentiation over time after induction of joint inflammation. Data correspond to the mean ± standard error of the mean (n = 6 horses). N/A, no differentiation performed given low white blood cell counts. *Significant difference from PIH 0 and 168 (*P *< 0.001). ^†^Significant difference from PIH 8 (*P *< 0.001).

### Synovial fluid mediators and markers

**Figure 2:**
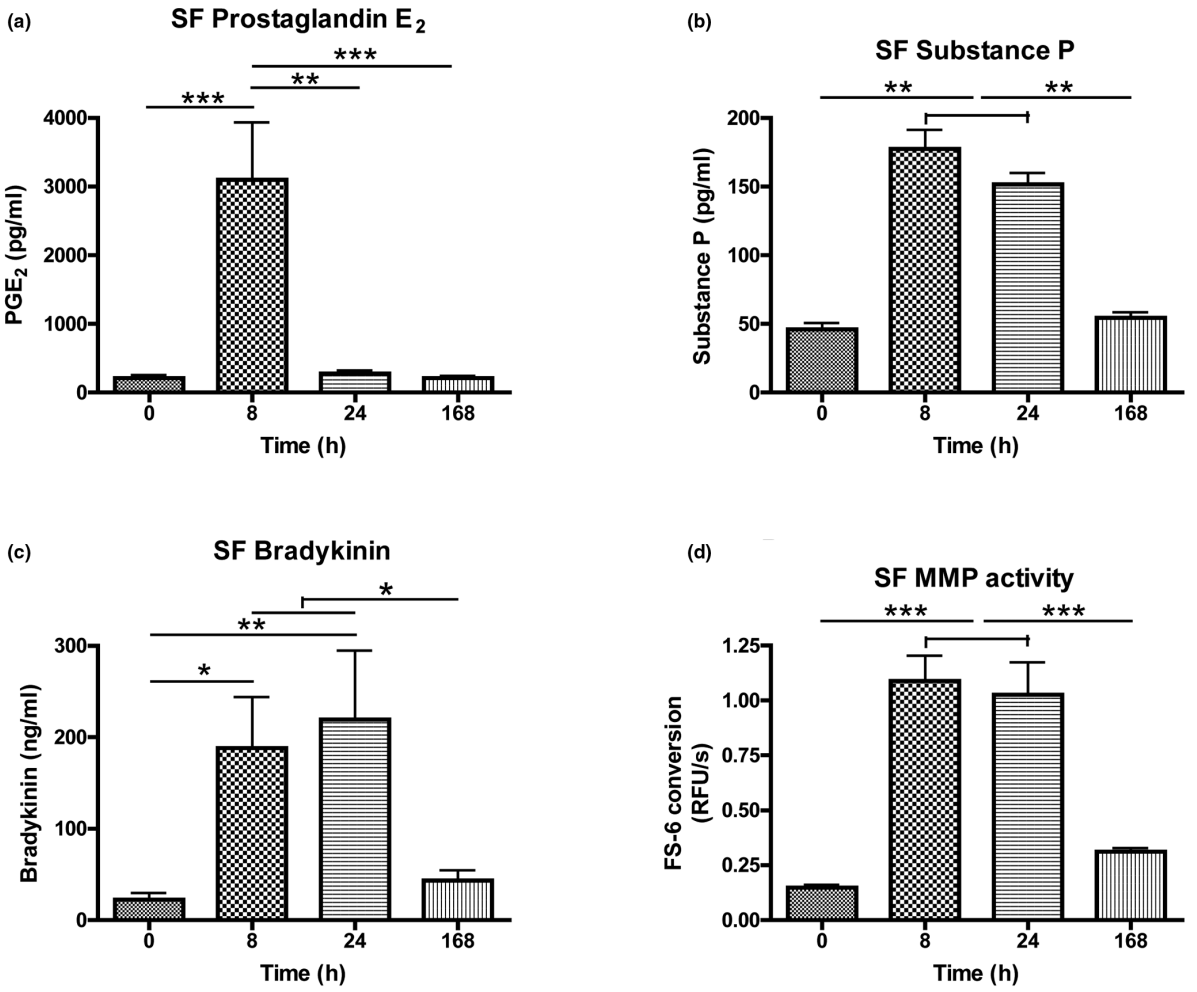
Synovial fluid inflammatory mediators and matrix metalloproteinase activity in inflamed joints. Synovial fluid (SF) levels of **(a) **prostaglandin E_2 _(PGE_2_), **(b) **substance P, **(c) **bradykinin, and **(d) **general matrix metalloproteinase (MMP) activity over time in inflamed intercarpal joints of horses (n = 6). Inflammation was induced at *t *= 0 by intra-articular injection of 0.5 ng *Escherichia coli *lipopolysaccharide. Data presented as the mean ± standard error of the mean. *P < 0.05, **P < 0.01, ***P < 0.001. RFU/s, relative fluorescence units/second.

**Figure 3:**
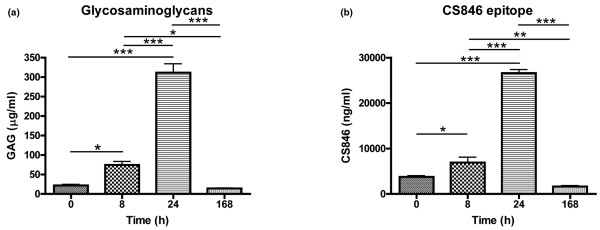
Synovial fluid levels of aggrecan turnover markers in inflamed joints. Synovial fluid concentrations of **(a) **glycosaminoglycans (GAG) and **(b) **chondroitin sulfate epitope 846 (CS846) over time in inflamed intercarpal joints of horses (n = 6). Inflammation was induced at *t *= 0 by intra-articular injection of 0.5 ng *Escherichia coli *lipopolysaccharide. Data presented as the mean ± standard error of the mean. *P < 0.05, **P < 0.01, ***P < 0.001.

**Figure 4:**
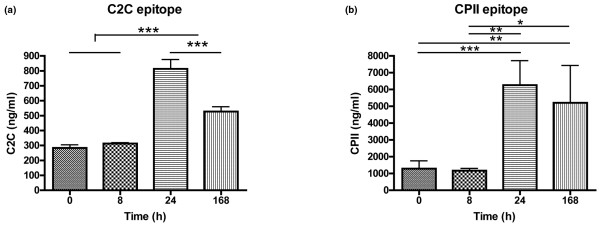
Synovial fluid levels of collagen II turnover markers in inflamed joints. Synovial fluid concentrations of **(a) **collagenase-cleavage neoepitope of type II collagen (C2C) and **(b) **carboxypropeptide of type II collagen epitope (CPII) over time in inflamed intercarpal joints of horses (n = 6). Inflammation was induced at *t *= 0 by intra-articular injection of 0.5 ng *Escherichia coli *lipopolysaccharide. Data presented as the mean ± standard error of the mean. *P < 0.05, **P < 0.01, ***P < 0.001.

Induction of inflammation led to a sharp rise in prostaglandin E_2 _at PIH 8, while substance P, bradykinin and MMP activity showed more sustained increases at PIH 8 and 24 (Figure 2). GAG release and the CS846 epitope showed parallel changes after LPS injection. Both were already significantly elevated by PIH 8, peaked at PIH 24, and returned to baseline levels by PIH 168 (Figure 3). For type II collagen, parallel profiles of putative catabolic (C2C) and anabolic (CPII) markers over time were also observed, but the time course differed from that seen for aggrecan markers in that collagen II markers rose later, at PIH 24, and were still elevated over baseline at PIH 168 (Figure 4).

## Discussion

Over the past decades, the importance of intra-articular inflammation in joint pain, joint effusion and progression of cartilage damage has become increasingly appreciated [2,33], as has the usefulness of synovial fluid biomarkers for monitoring disease progression or therapeutic response [5]. The magnitude and timing of the effects of active inflammation on SF biomarker levels, however – although acknowledged by many before – have received relatively little formal attention.

The inflammatory response elicited in the present study by intra-articular injection of 0.5 ng LPS was overt but also highly transient, and the absence of systemic signs of endotoxemia confirmed the local nature of LPS effects. The clinical response to LPS and changes in routine SF parameters closely paralleled those documented previously [21,22], confirming reproducibility of this model for induction of severe but transient joint inflammation.

We further characterized the induced inflammatory response through SF analysis of inflammatory mediators and pain-related (neuro)peptides. The observed increase in prostaglandin E_2 _was sharp and short-lived, which agrees with previous studies [19,34]. The involvement of bradykinin and substance P in this model is a novel finding; from studies on LPS-mediated effects in rodents, we hypothesized that LPS would indeed induce bradykinin and substance P release [35,36]. Owing to the descriptive nature of the present study and the known interactions between prostaglandin E_2_, substance P and MMP activity, we cannot determine to what extent changes in cartilage markers were due to each individual mediator. The observed increases in these mediators, however, do implicate each of them in the synovial inflammatory process, and they all may have contributed to the accompanying changes in cartilage turnover markers. While prostaglandin E_2 _and substance P are known actors in cartilage degradation in arthritic joints [3,37], the effects of bradykinin on articular cartilage remain largely unknown [38]. Certainly, our findings warrant further investigation of the involvement of each of these mediators and their receptors in altered cartilage turnover in arthritis.

The rise in prostaglandin E_2_, bradykinin and substance P in the first 24 hours coincided with an increase in MMP activity at PIH 8 and 24. The fluorogenic substrate used shows enhanced sensitivity for collagenase-mediated (MMP1, MMP8, MMP13) cleavage, but may also be cleaved by TNFα converting enzyme [26]. Unfortunately, activity assays that utilize capture antibodies for specific MMP subtypes have not yet been developed for use in the horse, so no inferences regarding activities of individual MMPs are justified.

LPS injection resulted in a swift and transient response of aggrecan turnover markers, both CS846 epitope and GAG release rising within 8 hours of LPS injection and returning to baseline after 1 week. This agrees with previously documented early increases in the release of aggrecan fragments from cartilage in response to injury or inflammatory stimuli [39,40]. Concentrations of the CS846 epitope in synovial fluid, proposed to reflect the turnover of novel aggrecan molecules [27,28], showed a strikingly similar time course to GAG levels following induction of inflammation. As both markers returned to baseline within 1 week, this inflammation-induced enhancement of aggrecan turnover seems to be both a fast and short-lived phenomenon.

A parallel course like that seen for aggrecan turnover markers was also evident for collagen II; however, the response of collagen II markers to LPS injection was delayed (at PIH 24) and persisted longer, with levels of both anabolic (CPII) and catabolic (C2C) markers still being elevated over baseline at PIH 168. This time lag between changes in aggrecan and collagen II markers in SF coincides with that seen in cartilage extracts in the rat mono-iodoacetate model of arthritis [41]. Concomitant with a rise in C2C at PIH 24 and 168, indicating inflammation-induced enhancement of collagen II cleavage [9], we found elevated concentrations of CPII. Increases in SF CPII levels have also been noted in human osteoarthritis and rheumatoid arthritis patients, as well as in animal models of osteoarthritis [7,17,29]. The increase in CPII following joint injury or with osteoarthritis development has generally been interpreted as a reparative response intended to mend damage to the collagen network. The current data indicate that a single transient inflammatory insult can also induce sustained changes in SF CPII levels over 1 week.

There are some limitations to the current study. The use of normal horses and a transient inflammatory stimulus may limit extension of the results to diseased individuals with more chronic inflammation. The choice of the LPS model for induction of joint inflammation was based on it having been well studied in the horse [19,21,22], showing reproducible local effects with accurate timing and quick recovery of joints. In addition, LPS is known to induce IL-1β and various other cytokines and mediators, such as TNFα and prostaglandin E_2_, implicated in naturally occurring arthritis [34,42-44]. Injection of LPS (or any other inflammatory stimulus) into a joint will elicit a release of inflammatory mediators from both the synovial membrane and the articular cartilage, which may affect cartilage integrity and thus SF marker levels. Apart from this indirect pathway, a direct effect of LPS on chondrocyte gene transcription cannot be excluded. No discrimination can be made between both effects on SF markers, but any significant direct LPS effect can be considered very small, given the dosage used [22].

The lack of visualization of the articular cartilage unfortunately precludes a direct appreciation of the effects of LPS-induced inflammation on the cartilage in the current set-up. Sacrifice of horses and analysis of cartilage at PIH 168, however, was beyond the scope of the current study, especially as we expected changes in marker levels to have returned to baseline by this time. In retrospect, it would have been interesting to extend the period of inflammation and SF collection, and to include a direct evaluation of the articular cartilage at baseline as well as at study completion, since this would have allowed us to assess the predictive value of SF marker concentrations for the level of cartilage damage incurred.

No sham-injected contralateral joints were included in the current study for comparison of the effect of repeated arthrocentesis alone on SF marker levels versus the effect of LPS; however, multiple studies have previously shown the effects of LPS on SF parameters to far exceed that of saline injection, and thus probably that of arthrocentesis alone [19,34]. Lastly, SF marker concentrations reported here were not corrected for dilution effects due to joint effusion using blood:SF urea ratios as suggested previously [12]. As LPS produced consistent joint effusion, the reported rises in marker concentrations will be an underestimate rather than an overestimate, and hence the observed trends and validity of conclusions drawn will be unaffected by correction.

## Conclusions

We have demonstrated pronounced effects of a single episode of joint inflammation on SF inflammatory mediators, MMP activity and cartilage biomarkers in healthy joints. In short, prostaglandin E_2_, substance P, bradykinin and MMP activity rise shortly after induction of inflammation; both markers of aggrecan and collagen II turnover increase in response to transient inflammation; putative anabolic and catabolic markers for each of these matrix components rise simultaneously; and the rise in collagen II turnover markers occurs slightly later than that of aggrecan markers, and persists longer. While the extension of these data to the clinical situation requires caution given the above limitations of the current model, the present study shows that a single event producing significant intra-articular inflammation may have a prolonged effect on SF concentrations of markers of cartilage turnover.

## Abbreviations

C2C: collagenase-cleavage neoepitope of type II collagen; CPII: carboxypropeptide of type II collagen; CS846: chondroitin sulfate epitope 846; ELISA: enzyme-linked immunosorbent assay; GAG: glycosaminoglycans; IL: interleukin; LPS: lipopolysaccharide; MMP: matrix metalloproteinase; PIH: post-injection hour; SF: synovial fluid; TNF: tumor necrosis factor.

## Competing interests

The authors declare that they have no competing interests.

## Authors' contributions

JCdG participated in the study design, carried out the experimental procedures, performed the mediator and marker (immuno)assays and drafted the manuscript. CHAvdL provided technical support with the synovial fluid analyses, performed the statistical analysis, and assisted in manuscript preparation. PRvW conceived of the study, participated in its design and coordination, and helped draft the manuscript. All authors read and approved the final manuscript.
